# Air traffic control activity increases attention capacity in air traffic controllers

**DOI:** 10.1590/S1980-57642010DN40300015

**Published:** 2010

**Authors:** Valdenilson Ribeiro Ribas, Hugo André de Lima Martins, Gutemberg Guerra Amorim, Renata de Melo Guerra Ribas, Cláudia Ângela Vilela de Almeida, Valéria Ribeiro Ribas, Carlos Augusto Carvalho de Vasconcelos, Murilo Duarte Costa Lima, Everton Botelho Sougey, Raul Manhães de Castro

**Affiliations:** 1 Doctor in Neuropsychiatry. Neuropsychiatry and Behavioral Sciences, Federal University of Pernambuco (UFPE), Third Integrated Center for Air Defense and Air Traffic Control (CINDACTA III), Recife PE, Brazil.; 2 Doctor in Experimental Pharmacology. Neuropsychiatry and Behavioral Sciences, Federal University of Pernambuco (UFPE), Third Integrated Center for Air Defense and Air Traffic Control (CINDACTA III), Recife PE, Brazil.; 3 Doctor Mental Health. Neuropsychiatry and Behavioral Sciences, Federal University of Pernambuco (UFPE), Third Integrated Center for Air Defense and Air Traffic Control (CINDACTA III), Recife PE, Brazil.; 4 Doctor in Medicine. Neuropsychiatry and Behavioral Sciences, Federal University of Pernambuco (UFPE), Third Integrated Center for Air Defense and Air Traffic Control (CINDACTA III), Recife PE, Brazil.; 5 Masters in Neurology. Neuropsychiatry and Behavioral Sciences, Federal University of Pernambuco (UFPE), Third Integrated Center for Air Defense and Air Traffic Control (CINDACTA III), Recife PE, Brazil.; 6 Masters in Neurology. Neuropsychiatry and Behavioral Sciences, Federal University of Pernambuco (UFPE), Third Integrated Center for Air Defense and Air Traffic Control (CINDACTA III), Recife PE, Brazil.; 7 Expert Sanitary Surveillance. Neuropsychiatry and Behavioral Sciences, Federal University of Pernambuco (UFPE), Third Integrated Center for Air Defense and Air Traffic Control (CINDACTA III), Recife PE, Brazil.; 8 Doctor Biological Science. Masters in Neuroscience. Neuropsychiatry and Behavioral Sciences, Federal University of Pernambuco (UFPE), Third Integrated Center for Air Defense and Air Traffic Control (CINDACTA III), Recife PE, Brazil.; 9 Masters in Neuropsychiatry. Neuropsychiatry and Behavioral Sciences, Federal University of Pernambuco (UFPE), Third Integrated Center for Air Defense and Air Traffic Control (CINDACTA III), Recife PE, Brazil.; 10 Doutor in Neuropsychiatry. Neuropsychiatry and Behavioral Sciences, Federal University of Pernambuco (UFPE), Third Integrated Center for Air Defense and Air Traffic Control (CINDACTA III), Recife PE, Brazil.

**Keywords:** air traffic controller, aeronautical information services, attention

## Abstract

Air traffic controllers simultaneously develop complex and multiple tasks in the course of their activities. In this context, concern is raised over the high level of attention needed by these professionals which can ultimately be affected by stress and fatigue.

**Objectives:**

The objective of this study was to assess attention level in air traffic controllers (ATCo).

**Methods:**

45 flight protection professionals were evaluated, comprising 30 ATCo, subdivided into ATCo with ten or more years in the profession (ATCo≥10, n=15) and ATCo with less than ten years in the profession (ATCo <10, n=15) and 15 aeronautical information services operators (AIS), subdivided into AIS with ten years or more in the profession (AIS≥10, n=8) and AIS with less than ten years in the profession (AIS <10, n=7), who were included as the control group. The digit symbol, d2 (the individual marks the letter d on a specific form containing 14 lines with 47 letters in each, maintaining focus on letter d followed by two dashes), forward digit span, backward digit span and PASAT (paced auditory serial addition test) attention tests were used. Kruskal-Wallis was used and data expressed as Median (Minimum and Maximum) with p<0.05.

**Results:**

The ATCo≥10 presented greater focus of attention, sustained attention, mental manipulation and resistance to interference capacity compared to the AIS≥10. Comparison of ATCo≥10 to the AIS<10 showed they presented only greater resistance to interference, and when compared to the ATCo<10 presented lower focus.

**Conclusions:**

The air traffic control activity after ten years may be associated with a high level of attention.

## Abstract (pt)

Os controladores de tráfego aéreo (CTA) desenvolvem em suas atividades tarefas complexas e múltiplas simultaneamente. Nesse contexto, surge a preocupação com a necessidade de um maior nível de atenção desses profissionais que pode ser afetada pelo estresse e fadiga.

**Objetivos:**

O objetivo deste trabalho foi avaliar o nível de atenção em controladores de tráfego aéreo (CTA).

**Métodos:**

Foram avaliados 45 profissionais de proteção ao vôo, sendo 30 CTA, subdivididos em CTA com dez ou mais anos na profissão (CTA≥10, n=15) e CTA com menos de dez anos na profissão (CTA<10, n=15) e 15 operadores de serviços de informações aeronáuticas (AIS), subdivididos em AIS com dez anos ou mais na profissão (AIS≥10, n=8) e AIS com menos de dez anos na profissão (AIS<10, n=7). Foram utilizados os testes de atenção *digit symbol*, d2 (o indivíduo deve marcar a letra d em um formulário específico, contendo 14 linhas com 47 letras cada, mantendo sempre o foco na letra d que aparece acompanhada de dois traços), *span* de dígitos em ordem direta e inversa e teste de dependência auditiva para adição em série (PASAT). Foi utilizado o Kruskal-Wallis e os dados expressos em mediana (mínimo e máximo), p<0,05.

**Resultados:**

Os CTA≥10 apresentaram maior foco de atenção, manutenção do foco, capacidade de manipulação mental e resistência à interferência, quando comparados aos AIS=10, mas quando comparados aos AIS<10 apresentaram somente maior resistência à interferência e quando comparados aos CTA<10, menor foco.

**Conclusões:**

A atividade de controle de tráfego aéreo após dez anos pode apresentar um alto nível de atenção.

Air traffic controllers, besides the problems caused by the inversion of shift work, have to carry out complex and multiple tasks simultaneously, such as controlling the navigation of several aircraft at the same time, coordinating with adjacent organs, and performing pre-planning, involving separation of leveled aircraft which are climbing or descending. In this context, a concern is raised over the high attention level required by these professionals, which may be affected by stress and fatigue.^1^

Some studies have demonstrated that stress affects the immune^2,3^ and nervous systems with regard to the cognitive functions^4^ including that of attention.^5^ Since 1980, behavior tests have been developed to evaluate attention and investigate the nervous circuits related to these processes. Results show that this was not a singular process, but consisted of different mechanisms which often complement one another.^6^ Among the several proposed models of attention, Mateer & Mapou (1996)^7^ proposed one that integrated all models that had gone before it. They established a kind of evaluation based on the division of attention into two main areas: deployment and encoding.^8^

Deployment refers to how well an individual can channel and focus attentional resources and includes arousal, focused and sustained attention. Arousal is evaluated by direct observation. Focused attention requires the individual to reject irrelevant information while concentrating on relevant input. It can be assessed by tasks that require quick scanning and identification of targets such as the digit symbol task (from Wechsler Intelligence Scale), the trail making test, the d2 (the individual marks the letter d on a specific form containing 14 lines with 47 letters each, focusing on letter d followed by two dashes), among other tests.^7, 8^

The second factor, capacity/encoding, refers to how well an individual can retain information in memory and then process it, even if distracted or required to divide attention among tasks. This can be tested using attention span (Forward Digit span), mental manipulation (Backward Digit span) and resistance to interference (PASAT-paced auditory serial addition test).^4,7^

The air traffic control profession can induce intense physical and mental burn out among these workers. Several studies have addressed this occupational stress, also called labor stress or organizational stress. Recently, a study was conducted investigating stress among Brazilian air traffic controllers.^9^ In the study, two investigation methods were used, a subjective method using the Lipp stress symptoms inventory, and an objective method based on biochemical markers, including cortisol concentration, nitric oxide level and monocyte phagocytosis rate. On both methods, the presence of stress was confirmed only among controllers with ten years or more in the profession.

Few studies have investigated the association between stress and attention. The present study verified the possible relationship among these factors given that research involving the Brazilian air traffic controller has a largely exploratory character, with the investigation instrument being essentially based on Mateer & Mapou’s (1996) theory.

Thus, the aim of the present study was to verify the forms of stress that affect cognitive levels of attention. However, although the use of neuropsychological evaluations has been growing considerably,^10^ the investigations into the association between stress and basic psychological processes remain scarce in the literature.^11^

## Methods

### Subjects

A total of 45 flight protection professionals were evaluated, comprising 30 air traffic controllers (ATCo) and 15 aeronautical information service operators (AIS) who were included as the control group. Notwithstanding shift work, the aeronautical information operators (AIS) perform a different role to that of controllers. The specification of this function will be presented below. The professionals belong to the Third Integrated Center of Air Defense and Air Traffic Control (CINDACTA III) and the Aeronautical Command (COMAER) in Recife/PE, Brazil. More specifically, the air traffic controllers were from the area control Center (ACC). The subjects were submitted to the evaluations at the CINDACTA III, under standard conditions at 08:00a.m., at the beginning of their shifts in a building with central air-conditioner, maintaining a temperature of 22º±2ºC. The professionals were informed about the test on the previous day and all subjects agreed to sleep at 08:00 p.m. the day before the attention tests. Male air traffic controllers were included in the research whereas inactive or female controllers were excluded because of their small number.

#### Air traffic control activity specifications

Air traffic control is rendered by three units of control called: Tower (TWR), Approach Control (APP) and Area Control Center (ACC), which as a convention, even in Brazil, are acronyms from the English language. The controllers from the Tower are responsible for traffic during landing and take-off situations, and also for the movements of people and vehicles in the maneuvering area, and monitor the tracks and roads used for local circulation. In terms of vertical division, the tower has jurisdiction on all traffic flying at altitudes up to 2,000 feet.

**Figure1:**
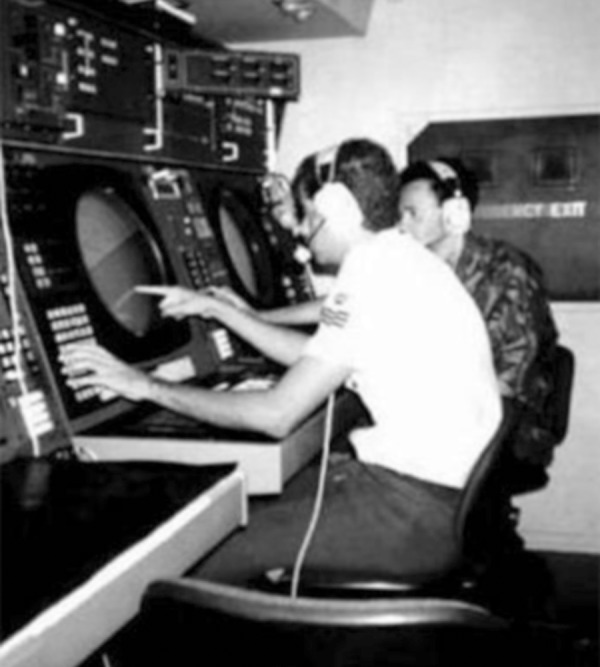
Approach Control (APP), Natal, Rio Grande do Norte, Brazil. Natal APP belongs to the third integrated center of air defense and air traffic control (CINDACTA III) of the Aeronautical Command (COMAER).

Approach Control (APP)(Figure 1) is the entity responsible for the intermediary phase of the flight. In large capitals, areas are usually mapped out called terminals (TMA) that consist of route letters and manuals available to the airmen. These areas cover a lateral approach to a distance of 40 nautical miles (NM) or slightly over. APP has jurisdiction over traffic that flies at between 2,000 and 14,000 feet. The Area Control Center (ACC) usually controls a much larger air space than the above-mentioned organs. ACC Recife, for example, controls all aircraft in the whole Northeast of Brazil flying at 14,000 feet or above. The AIS, despite also having shift work, carries out a very different service to the air traffic controllers. AIS organizes publications that involve flight protection in the form of Aeronautics Command Instructions (ICAs), among them ICA 100-12, describing air traffic regulation throughout Brazil.

#### Control group activity specifications (Aeronautical Information Operators – AIS)

The AIS operators organize publications that involve flight protection, such as Aeronautics Command Instructions (ICAs). Among these ICAs is ICA 100-12, which is related to the nationwide air traffic rules. Besides this type of activity, they also guide the pilots and the operational flight dispatchers (DOV) during the completion of flight plans and give them important notices about flight security called *Notice to Airman* (NOTAM). These NOTAM contain information about restricted, dangerous or prohibited areas relating to air combat training by fighters from the Brazilian Air Force, Army and Navy, all occupying defined areas of the Brazilian air space, where pilots will not be able to fly or overfly and are obligated, according to ICA 100-12, to be aware of all NOTAMs before departure.

#### Groups

The subjects were divided into four groups: aeronautical information service operators AIS (control group), subdivided into AIS male operators in the 30-45 age group with ten years or more in the profession, (AIS≥10, CONTROL 1), n=8; AIS male operators in the 18-29 age group with less than ten years in the profession (AIS<10, CONTROL 2), n=7; air traffic controllers ATCo male controllers in the 30-45 age group with ten years or more in the profession (ATCo≥10), n=15 and ATCo male operators in the 18-29 age group with less than ten years in the profession (ATCo<10), n=15. All subjects held university degrees.

The decision to elect the 10-year experience as the parameter for the data collection and group divisions was based on an earlier doctoral thesis.^9^ In the cited study, information was collected by questionnaire that contained data on greater prevalence of headache, anxiety, depression, hypertension, infections and viruses after ten years in the profession.

### Attention evaluation

The attention assessment was applied by a psychologist, (VRR), in accordance with the Brazilian Federal Council of Psychology guidelines.

#### Deployment factor

Digit symbol and d2 test application.

The digit symbol tests requests the correct correspondence of the numbers from 1 to 9 and their respective symbols within 1 minute and 30 seconds; while in the d2 the individual has to mark the letter d on a specific form containing 14 lines with 47 letters each, maintaining focus for d2. According to the previous explanation, the position of the letter “d “s can be differentiated with single or double lines, above or below the letter.

#### Attention capacity factor

Forward Digit Span – Backward Digit Span, and Paced Auditory Serial Addition Test (PASAT).

Forward Digit Span-First with a sequence of 2 digits and then increasing progressively. The subject must repeat the sequence correctly. This is an indirect form of measuring the amount of information that the subject is able to retain.

Backward Digit span is similar to the Forward Digit span, only the subject has to repeat the digit sequence in reverse order. For example, with 4-3-7, the individual should repeat 7-3-4, and so forth. The digit sequence increases and the subject continues repeating in reverse order. Besides retaining information in the memory, the subject must also mentally manipulate the information.

PASAT – This is a test that verifies the capacity for mental manipulation of information and can also measure capacity for resisting interference. This resistance to interference is the third sector that must be evaluated in order to verify attention capacity. In this test, the subject has to add a dictated sequence of numbers. The examiner says the first number then the second, and from this second number, the subject has to add it to the previous one. For example, 4, 7=11. When the next number is given, the subject must add it to the last number of the previous sequence, which was 7, rather than to the result which was 11. The subject must then be able to maintain this latest number in the series while discarding the previous sum. This test assesses the subject’s capacity to mentally manipulate information and tests resistance to interference. In this study, resistance to interference was not assessed.

### Data analysis

The data found in the attention tests, applied individually, were analyzed by Kruskal-Wallis one way analysis of variance by ranks test, where all pairwise multiple comparison procedures used Dunn’s method, and data was expressed as Median (Minimum and Maximum) with p<0.05.

## Results

### Focused Attention evaluation-Digit Symbol

ATCo≥10 presented greater focus of attention 62(39-65) compared to the AIS≥10 47(38-64, p<0.05*). The ATCo<10 presented greater focus of attention 65(56-79, p<0.05#) compared to the ATCo≥10 62(39-65), to the AIS<10 60(53-63) and AIS≥10 47(38-64, p<0.05#)(Table 1).

**Table 1:** Attention evaluation.

Focused - Sustained attention - Mental manipulation - Resistance to interference				
	**Digit symbol**	**d2 Test**	**Reverse digit span **	**Pasat**
AIS≥10 (N=8)	47 (38-64)	405 (333-444)	6 (3-7)	8 (5-14)
AIS<10 (N=7)	60 (53-63)	475 (415-492)	8 (5-10)	9 (7-10)
ATCo<10 (N=15)	65 (56-79)^#^↑	489 (401-570)^#^↑	9 (4-11)^#^↑	14 (9-14)^#^↑
ATCo≥10 (N=15)	62 (39-65)*↑	480 (298-556)*↑	9 (5-9)*↑	15 (6-16)*↑

45 flight protection professionals were evaluated, comprising 30 ATCo, subdivided into ATCo with ten or more years in the profession (ATCo≥10, n=15) and ATCo with less than ten years in the profession (ATCo <10, n=15), along with 15 aeronautical information services operators (AIS), subdivided into AIS with ten years or more in the profession (AIS≥10, n=8) and AIS with less than ten years in the profession (AIS<10, n=7), which comprised the control group. The Kruskal-Wallis test was used, and all pairwise multiple comparison procedures were performed with Dunn's method and data was expressed as Median (Minimum and Maximum) with p<0.05^*#^.

### Sustained attention – *d2* Test

ATCo≥10 presented longer sustained attention 480(298-556) compared to the AIS≥10 405 (333-444, p<0.05*). ATCo<10 presented longer sustained attention 489(401-570) compared to AIS≥10 405 (333-444, p<0.05#)(Table 1).

### Mental manipulation – backward digit span

ATCo≥10 presented greater mental manipulation capacity 9(5-9) compared to the AIS≥10 6(3-7, p<0.05*). ATCo<10 presented greater mental manipulation 9(4-11) compared to the AIS≥10 6(3-7, p<0.05#)(Table 1).

### Resistance to interference – PASAT

ATCo≥10 presented greater resistance to interference capacity 15(6-16) compared to the AIS≥10 8(5-14, p<0.05*) and to the AIS<10 9(7-10, p<0.05*). ATCo<10 presented greater resistance to interference 14(9-14) compared to the AIS≥10 8(5-14, p<0.05^#^) and to the AIS<10 9(7-10, p<0.05^#^)(Table 1).

## DISCUSSION

The working attention level after 8 (eight) hours of work was not evaluated in this study, which instead investigated whether chronic stress affects the cognitive system after many years of work. The hypothesis that motivated this study stems from the notion that the stress experienced by air traffic controllers resembles post-traumatic stress. According to Meewisse et al. (2005 *apud* Koso & Hansen, 2006)^12^ who carried out a study on 124 survivors in Holland, after only months, post-traumatic stress causes attention dysfunction in affected individuals.^12^

Thus, in this study, attention was evaluated revealing an increase in the focus of attention, sustained attention, mental manipulation and resistance to interference in both air traffic controllers with less, and more, than ten years in the profession, versus controls. These results corroborate Rodrigues’ findings in 2010^13^ who described an increase in the focus of attention, and also those of Menezes et al. (2009)^8^ for mental manipulation. However, our results contrast those of Koso & Hansen (2006),^12^ Borges (2007)^5^ and Guerra-Ribas (2009 *apud* Ribas, 2009)^4^ relating focus of attention and resistance to interference aspects. However, it is important to point out the methodological differences and similarities among these studies.

The subjects evaluated in the studies by Rodrigues et al. (2010) and Menezes et al. (2009) were male pupils in the 9 to 12 year age bracket who participated in a cultural movement and students whose parents graduated from college, who had reading habits. The similarities lie in the theoretical line of the methodology employed, because both studies used Mateer and Mapou’s (1996) theory of attention and, possibly in the cerebral perfusion pattern alteration with blood flow increase that is typically observed on neuroimaging structural exam after physical exercise,^14^ during reading^15^ and in individuals that make persistent use of cognitive faculties, whether during work activity or in day-to-day tasks such as readings, computer use and others.^8^

The blood flow displacement seen in cognitive activities has been confirmed in recent years in neuroimaging techniques used in the investigation of the workings of the human brain. The most traditional methods are positron emission tomography-PET and single photon emission computed tomography-SPECT. Both techniques allow the construction of three-dimensional maps of brain activity from the emission of gamma rays for a tracer marked with radioactive isotope administered intravenously or by inhalation.^16^

The methodological differences among the studies with conflicting results were that the study by Koso & Hansen (2006) involved victims of sexual abuses and by Borges (2007) was in war veterans, both with Post-traumatic Stress disorders, while the study by Guerra-Ribas (2009) involved those who worked with food preparation (cooks, butlers) from the public hospital of Recife. In the first two studies cited above, there seems to be a resemblance to the present study in terms of the form of expression of stress, since in case of long-term stress and post traumatic stress disorders-PTSD, the performance of attention may be related to frontal cortex dysfunction and its connections to the limbic system^17,18^ especially, in air traffic controllers that were longer in the profession, who presented long-term stress which was predominantly psychological.

Besides showing similarity in the psychological expression of stress among these studies, day-to-day activities as well as the labor function seem to be an essential characteristic in attention development. However, the subjects assessed by the authors did not perform any activity involving continuous use of cognitive faculties in the manner that air traffic controllers do.

Koso & Hansen (2006) studied students, Borges (2007) veteran soldiers, and Guerra-Ribas investigated workers that make little use of cognitive structures as a working tool, as they were workers that perform mainly manual labor. Similarly, little cognitive use occurs in the execution of ready prescriptions for patients of a public hospital in Recife, in contrast with air traffic controllers whose work tools are language, reasoning, quick thinking, memory, concentration, attention and constant fear of making a mistake.

It is clear that further studies with a longitudinal design containing questions related to these professionals’ childhood can contribute toward a better comprehension of long-term stress effects. The literature has shown that traumatic events in childhood, characterized chronically, may interfere in the process of maturation and cerebral organization, due to chronic hyper activation of the neural systems of responses to stress.

Although there is no dichotomy between the organism and thought, besides the possible physiological interference during the maturation of the nervous system caused by minor traumas in childhood, there is also the form of conception of subject and world constituted in the personality structure acquired from each air traffic controller’s relationship with their father, mother and children that may cause irritation to one professional yet not to others, such as their identification with the administrative model, the rules of which they comply with.

The results of this study lead us to consider that the similarity between air traffic controllers’ stress and post-traumatic stress may only occur at a cognitive level, but not at a neuro-cognitive level, because according to Meewisse et al. (2005 *apud* KOSO & HANSEN, 2006), post-traumatic stress affects attention performance.^13^

In this sense, the results evidencing increased attention in Brazilian air traffic controllers in this study seem to be directly related to constant training and the complexity of the daily activity per se, and are not due to stress.

Although this study was rigorously conducted with close attention to method, these results cannot be generalized to the 3,000 (three thousand) Brazilian flight controllers in the profession, because this study, although presenting reliable results was an exploratory case study. However, these results warrant the performing of further studies involving a larger number of subjects.
